# Supplementary material for: Assessing the effectiveness of newborn resuscitation training and skill retention program on neonatal outcomes in Madhesh Province, Nepal
Source: PLOS Glob Public Health. 2022 Oct 21;2(10):e0000666. doi: 10.1371/journal.pgph.0000666 (PMC10022377; doi:10.1371/journal.pgph.0000666)
Supplement: Attachment — Submitted filename: Response to Reviewers.docx [file pgph.0000666.s002.docx]

**Response to Reviewers**

August 25, 2022

Dear Editors and Reviewers,

Thank you very much for inviting us to submit a revised draft of our manuscript titled, “Assessing the effectiveness of newborn resuscitation training and skill retention program on neonatal outcomes in Madhesh Province, Nepal” to PLOS Global Public Health. We are also thankful to you and the reviewers for insightful comments to our manuscript. The reviewers’ comments were very helpful in strengthening our paper.

We are delighted to resubmit our article for further consideration. We have carefully considered the reviewers’ feedback and suggestions in the revised manuscript. We hope that our edits and the point-by-point response to the reviewers’ comments we provide below appropriately address all the issues raised. We also hope that the revisions meet the expectation requirements necessary for you to accept our manuscript for publication in PLOS Global Public Health.

Thank you,

Dr. Robert B Clark, on behalf of all authors.

**Editor Suggestions:**

1. **Please update your online Competing Interests statement. If you have no competing interests to declare, please state: “The authors have declared that no competing interests exist.”**

Thank you for noting this. As you suggested, we have updated our online Competing Interests Statement.

2. **Please provide a complete Data Availability Statement in the submission form. If your research concerns only data provided within your submission, please write “All data are in the manuscript and supporting information files.” as your Data Availability Statement.**

We sincerely appreciate your suggestion. We have provided a complete Data Availability Statement in the submission form as suggested.

3. **We have noticed that you have uploaded Supporting Information files, but you have not included a list of legends. Please add a full list of legends for your Supporting Information files after the references list.**

We agree with your assessment. We have incorporated your comment by adding a full list of legends for our Supporting Information files (p. #31, line #651).

**Response to Reviewers**

**Reviewer 1 comments:**

**I am glad to review this paper entitled “Assessing the effectiveness of newborn resuscitation training and skill retention program on neonatal outcomes in Madhesh Province, Nepal.” I would like to congratulate the team for doing this project to improve the quality of care in resource limited settings and identifying the area where neonatal resuscitation programme was actually required. Decreasing neonatal mortality is really a great challenge in resource limiting settings. This research has utilized evidenced based strategies and implemented a cost-effective strategies/programmer to overall improve the neonatal outcomes in this province. This paper is well written and results are well expressed.**

**Though this study was not a clinical study, some queries-**

1. **Is there data regarding baseline characteristics of neonates in baseline period and endline period? As we know, there are various factors that can affect outcome of neonates within 24 hours and after 24 hours like maternal factors and fetal factors.**

Thank you very much for raising this very important point. We agree with your view that neonatal outcome within and after 24 hours of life is affected by various maternal and fetal factors such as maternal education, socio-economic status, quality of antenatal care, neonatal sepsis and prematurity among others. However, the newborn resuscitation training, scale-up and skill retention program focused on tracking minimal facility-level metrics to monitor project. Since the program utilized these routinely collected facility-level metrics, we are limited in analyzing the role of various maternal and fetal factors that may have contributed to the findings of our study.

We have addressed this comment by adding the use of routinely collected data as one of the limitations in the discussion section. (p.#26, line #500-506)

**2. It’s better to present the data in Table 4 as both number values and statistically significant observation for better understanding of results.**

Table 4 has been adjusted by providing baseline mean, endline mean and p-value for each outcome by facility type.

**3. Minor queries: Abstract section line no 35, please mention the full form of LDS Charities here as well.**

We agree with you. We have added the full form of LDS Charities in the abstract section (p. #2, line #35)

**Reviewer 2 comments:**

**Yes, the manuscript is well written, clear and correct in most of its aspect and unambiguous. However, there were few grammatical errors which needs to be corrected and few numerical errors along with few minor changes, which I have attached as an attachment.**

Experienced scholarly writers who are native speakers have proofread the manuscript again and grammatical as well as numerical errors are corrected, as suggested. We have also completed minor changes as suggested and a few other adjustments. Please refer to the change-tracked copy, where all changes are noted.

Reviewer 2 very thoughtfully included a number of comments and suggestions on the manuscript which was returned to us for review. We have incorporated the recommendations into the manuscript, as changes or additions in text, in all instances except for three. In two instances the information requested was not collected due to the nature of limited program monitoring. In one instance, the terminology chosen was consistent with the source documents. Additional comments and specific details show as responses to comments on the Revised Manuscript with Tracked Changes document.
